# Evaluation of a fluorescence *in situ* hybridization (FISH)-based method for detection of SARS-CoV-2 in saliva

**DOI:** 10.1371/journal.pone.0277367

**Published:** 2022-11-08

**Authors:** Gerrit G. Tamminga, Gijsbert J. Jansen, Marit Wiersma

**Affiliations:** Biotrack, Leeuwarden, The Netherlands; University of Georgia, UNITED STATES

## Abstract

The use of a non-invasive fluorescence *in situ* hybridization (FISH)-based method on saliva for the detection of SARS-CoV-2 is evaluated in a proof-of-concept study and thereafter utilized in an outpatient setting with the Biotrack-MED® analyzer. For a proof-of-concept study, saliva samples were obtained from 28 persons with mild or moderate COVID-19-related symptoms who were tested RT-PCR positive or negative for SARS-CoV-2. In an outpatient setting, 972 individual saliva samples were utilized. All saliva samples were FISHed with a Cy3-labeled SARS-CoV-2-specific DNA probe and were analyzed manually by fluorescence microscopy (proof-of-concept) or with the SARS-CoV-2 application of the Biotrack-MED® analyzer, a semi-autonomous multi-sample filter cytometer. The proof-of-concept study showed a sensitivity of 96.0% and a specificity of 98.5% and is therefore comparable to the RT-PCR analysis of nasopharyngeal swabs. The outpatient setting showed a sensitivity of 90.9% and a specificity of 94.5% and seems therefore a valid assay for the detection of SARS-CoV-2 in individuals that are healthy, mild or moderate symptomatic. In conclusion, the method evaluated in this study, the FISH-based SARS-CoV-2 application of the Biotrack-MED® analyzer, is a sensitive and reliable assay for the detection of SARS-CoV-2 in the general population.

## Introduction

Coronavirus disease of 2019 (COVID-19), caused by the Severe Acute Respiratory Syndrome Coronavirus-2 (SARS-CoV-2), is a severe acute respiratory syndrome causing morbidity and mortality worldwide. In March 2020, the WHO declared COVID-19 as a pandemic [1]. By half October 2022, more than 621.797.00 confirmed infections and approximately 6.545.560 deaths are reported worldwide [2]. To stop the spread of the virus, countries have taken several control measures, including social distancing, restriction of group gatherings and lockdowns. Due to the pandemic and these pandemic-related measures, there is a major impact on health(care) and economy [3]. Early detection of infected persons will aid in stopping the COVID-19 pandemic.

At the moment, the reverse transcription polymerase chain reaction (RT-PCR), detecting the presence of intra- and extracellular viral nucleotides, is accepted by the WHO, the FDA and other national health organizations as being the ‘gold standard’ for detection of SARS-CoV-2 RNA. However, in the early stages of COVID-19, viral load may be below the detection limit of the RT-PCR, resulting in a false-negative result [4]. The RT-PCR is performed on respiratory samples of nasopharyngeal and/or oropharyngeal swabs, an invasive and laborious method that requires trained personnel, causes discomfort to the tested person and causes an infection risk for the trained personnel [5,6]. A less laborious method is the antigen test. Although this method shows a lower time to result, there appears to exist an alarming lack of sensitivity resulting in lower sensitivity and specificity scores. A person with a large load of SARS-CoV-2 will be tested positive with an antigen test, but a person with a small load of SARS-CoV-2 will not be detected and will be passed as negative, even though the person is contagious [7,8]. Therefore, a less invasive method that is also able to detect COVID-19 in the early stages is needed.

One option for a less invasive method is the use of saliva as a matrix for SARS-CoV-2 detection. Besides being less invasive, there is less aversion, the costs are lower and no trained personnel is necessary for sampling, thereby reducing the infection risk and increasing the number of individuals that can be tested [9]. Importantly, the salivary glands and saliva are both thought to be a SARS-CoV-2 infection site and transmission route in (pre-)symptomatic and asymptomatic individuals [10]. This is supported by the WHO, which states that SARS-CoV-2 spreads through aerosols from the mouth or nose of an infected individual [11]. Several studies have compared the results of RT-PCR on saliva versus nasopharyngeal swab and found that they correlate well in several settings (outpatient (healthy, asymptomatic, symptomatic) and inpatient), with saliva detecting sometimes up to 10% more positive individuals [10,12–16]. Prior to the COVID-19 pandemic, other respiratory viruses, including influenza A, influenza B and respiratory syncytial virus (RSV), have been detected in saliva with high sensitivity [17,18]. Saliva testing for the detection of SARS-CoV-2 is currently implemented in several countries, including South Korea, Japan, USA and Germany [12].

Previously, we described a pilot study to detect SARS-CoV-2 in saliva intracellularly by means of a fluorescence *in situ* hybridization (FISH)-based method [19]. Here, we first describe the manual evaluation of this method in mild to moderate symptomatic individuals as a proof-of-concept. Secondly, we show how this method operates automatically in an outpatient setting, utilizing the Biotrack-MED® analyzer as a sample reading platform.

## Methods

### Study population—Proof-of-concept

This study population consisted of 28 persons (88% men and 12% women) in the age of 18–70 year. Inclusion criteria were: ≥ 18 years of age, up to 2 days mild to moderate symptomatic (Table 1) at the time of saliva sampling and samples were taken between 1-10-2020 and 1-12-2020. In addition, all included persons have been tested for SARS-CoV-2 by a local Dutch Municipal Health Services by RT-PCR of a nasopharyngeal swab ± 1 day before or after the saliva sample collection. Ten persons (P1-P10) were SARS-CoV-2 RT-PCR positive, of whom 80% presented with mild symptoms and 20% with moderate symptoms. Eighteen persons (N1-18) were SARS-CoV-2 RT-PCR negative and all of these persons presented with mild symptoms.

**Table 1:** Symptoms of mild- and moderate-COVID-19-symptomatic patients.

Mild-symptomatic	Moderate-symptomatic
Nasal congestion	Nasal congestion
Runny nose	Runny nose
Sore throat	Sore throat
Low fever	High fever
Dry cough	Deep cough
Fatigue	Fatigue
Headache	Headache
Loss of taste/smell	Loss of taste/smell
Nausea/vomiting	Nausea/vomiting
Diarrhea	Diarrhea
	Chills
	Muscle pain
	Shortness of breath

### Study population—Outpatient setting

This study population consisted of 972 persons (83% men and 17% women) in the age of 18–70 year, whom used the commercial services of Biotrack, Leeuwarden to detect the presence of SARS-CoV-2 in saliva between December 2020 and April 2021. These persons were either healthy, mild symptomatic or moderate symptomatic. Of these persons, no data was available whether they were RT-PCR tested by a local Dutch Municipal Health Services.

### Ethics

All persons included used the commercial services of Biotrack, Leeuwarden to detect the presence of SARS-CoV-2 in their saliva and signed and approved for the use of their archived and fully anonymized saliva samples for research purposes. Since the saliva samples were anonymized diagnostic surplus material, no approval from the Medical Research Ethics Committee was needed, according to the Code of conduct for responsible use (Foundation Federation of Dutch Medical Scientific Societies).

### Saliva sample collection

Saliva was obtained with a Biotrack saliva sample kit, containing instructions and a sample collection tube filled with 5 ml Biotrack Preservation Fluid. First, the person rinsed his/her mouth with mineral water and waited for 5 minutes. Next, the person added 1:1 saliva (5 ml) to the sample collection tube. After 10 minutes at room temperature, the saliva samples were fixed.

### Pooled saliva samples–proof-of-concept

As the proof-of-concept study population consisted only of 28 persons (P1-P10 and N1-N18), the saliva samples of this study population were pooled to increase the dataset in order to reduce the risk of overfitting in the machine learning [20,21]. of the SARS-CoV-2 application of the Biotrack-MED® analyzer (see data acquisition and interpretation). P1-P10 were pooled 1:1 or 1:2 (v/v) according to the scheme of Table 2 resulting in 100 saliva samples. N1-N18 were pooled 1:1 or 1:2 (v/v) according to the scheme of Table 3 resulting in 324 saliva samples. In addition, the original samples (0 in Tables 2 and 3) were also measured.

**Table 2:** Scheme of the pooling of the saliva samples of the SARS-CoV-2 RT-PCR positive persons (v/v).

P	1	2	3		4	5	6	7	8	9	10
1	0	1:1	1:1		1:1	1:1	1:1	1:1	1:1	1:1	1:1
2	1:2	0	1:1		1:1	1:1	1:1	1:1	1:1	1:1	1:1
3	1:2	1:2	0		1:1	1:1	1:1	1:1	1:1	1:1	1:1
4	1:2	1:2	1:2		0	1:1	1:1	1:1	1:1	1:1	1:1
5	1:2	1:2	1:2		1:2	0	1:1	1:1	1:1	1:1	1:1
6	1:2	1:2	1:2		1:2	1:2	0	1:1	1:1	1:1	1:1
7	1:2	1:2	1:2		1:2	1:2	1:2	0	1:1	1:1	1:1
8	1:2	1:2	1:2		1:2	1:2	1:2	1:2	0	1:1	1:1
9	1:2	1:2	1:2		1:2	1:2	1:2	1:2	1:2	0	1:1
10	1:2	1:2	1:2		1:2	1:2	1:2	1:2	1:2	1:2	0

**Table 3:** Scheme of the pooling of the saliva samples of the SARS-CoV-2 RT-PCR negative persons (v/v).

N	1	2	3	4	5	6	7	8	9	10	11	12	13	14	15	16	17	18
1	0	1:1	1:1	1:1	1:1	1:1	1:1	1:1	1:1	1:1	1:1	1:1	1:1	1:1	1:1	1:1	1:1	1:1
2	1:2	0	1:1	1:1	1:1	1:1	1:1	1:1	1:1	1:1	1:1	1:1	1:1	1:1	1:1	1:1	1:1	1:1
3	1:2	1:2	0	1:1	1:1	1:1	1:1	1:1	1:1	1:1	1:1	1:1	1:1	1:1	1:1	1:1	1:1	1:1
4	1:2	1:2	1:2	0	1:1	1:1	1:1	1:1	1:1	1:1	1:1	1:1	1:1	1:1	1:1	1:1	1:1	1:1
5	1:2	1:2	1:2	1:2	0	1:1	1:1	1:1	1:1	1:1	1:1	1:1	1:1	1:1	1:1	1:1	1:1	1:1
6	1:2	1:2	1:2	1:2	1:2	0	1:1	1:1	1:1	1:1	1:1	1:1	1:1	1:1	1:1	1:1	1:1	1:1
7	1:2	1:2	1:2	1:2	1:2	1:2	0	1:1	1:1	1:1	1:1	1:1	1:1	1:1	1:1	1:1	1:1	1:1
8	1:2	1:2	1:2	1:2	1:2	1:2	1:2	0	1:1	1:1	1:1	1:1	1:1	1:1	1:1	1:1	1:1	1:1
9	1:2	1:2	1:2	1:2	1:2	1:2	1:2	1:2	0	1:1	1:1	1:1	1:1	1:1	1:1	1:1	1:1	1:1
10	1:2	1:2	1:2	1:2	1:2	1:2	1:2	1:2	1:2	0	1:1	1:1	1:1	1:1	1:1	1:1	1:1	1:1
11	1:2	1:2	1:2	1:2	1:2	1:2	1:2	1:2	1:2	1:2	0	1:1	1:1	1:1	1:1	1:1	1:1	1:1
12	1:2	1:2	1:2	1:2	1:2	1:2	1:2	1:2	1:2	1:2	1:2	0	1:1	1:1	1:1	1:1	1:1	1:1
13	1:2	1:2	1:2	1:2	1:2	1:2	1:2	1:2	1:2	1:2	1:2	1:2	0	1:1	1:1	1:1	1:1	1:1
14	1:2	1:2	1:2	1:2	1:2	1:2	1:2	1:2	1:2	1:2	1:2	1:2	1:2	0	1:1	1:1	1:1	1:1
15	1:2	1:2	1:2	1:2	1:2	1:2	1:2	1:2	1:2	1:2	1:2	1:2	1:2	1:2	0	1:1	1:1	1:1
16	1:2	1:2	1:2	1:2	1:2	1:2	1:2	1:2	1:2	1:2	1:2	1:2	1:2	1:2	1:2	0	1:1	1:1
17	1:2	1:2	1:2	1:2	1:2	1:2	1:2	1:2	1:2	1:2	1:2	1:2	1:2	1:2	1:2	1:2	0	1:1
18	1:2	1:2	1:2	1:2	1:2	1:2	1:2	1:2	1:2	1:2	1:2	1:2	1:2	1:2	1:2	1:2	1:2	0

### Fluorescence in situ hybridization (FISH)

For the FISH procedure of the proof-of-concept, the Biotrack COV19 Probe directed at the antisense genomic RNA of the nucleocapsid gene of SARS-CoV-2 (+; sense probe, Cy3 labeled, 24 nucleotides, GC: 41.7%, Tm: 60.2°C, *in silico* specificity of 100%, LoD of 1.4 copies of viral RNA per 1 μl sample) [19], the Biotrack Wash Buffer (both part of the BTMED COV19 test kit) and microscopic slides with 10 reaction wells (VWR, The Netherlands) were used. The saliva samples were mixed 1:2 (v:v) with the Biotrack COV19 Probe, whereafter the mixtures were hybridized at 50°C for 1.5 hour. Next, 1 ml Biotrack Wash Buffer was added to the suspension and subsequently incubated during 30 min. at 60°C. Finally, the suspension was centrifuged (10 min. at 800 x g), the supernatant removed and the pellet resuspended in 50 μl Milli-Q. An aliquot of 10 μl from every saliva sample was added to a well on the microscopic slide, dried for 10 min. at 50°C and mounted with Fluoroshield (VWR, The Netherlands).

For the FISH procedure for the outpatient setting, a Biotrack proprietary test kit (BTMED COV19 test kit) was used, consisting of the Biotrack COV19 Probe, Biotrack Wash Buffer and filter cups. The same procedure as above was followed, only an aliquot of 30 μl from every saliva sample was added into a plastic sample container (a filter cup), which was fitted into an analysis disk, dried for 25 min. at 50°C and placed into the Biotrack-MED® analyzer for autonomous microscopic reading. The time-to-result for the whole procedure (FISH and Biotrack-MED® analysis) was approximately 3 hours for 18 samples, in which the FISH analysis is approximately 2,5 hours and the machine analysis (Biotrack-MED®) approximately 30 minutes.

### Data acquisition and interpretation

The fluorescently labeled saliva samples of the proof-of-concept setting were manually analyzed by utilizing a Leica DM2500 fluorescence microscope equipped with a Leica EL6000 mercury lamp and a Leica DFC450C camera. Images with a magnification of 400x were taken using the software Leica application suite V4.20. The images were blinded and manually analyzed independently by three experienced researchers in a binary fashion, rendering the result as positive as at least one white blood cell contained 2 or more fluorescent spots. Thereafter, the images were used to train the multi-layer neural network of the SARS-CoV-2 application of the Biotrack-MED® analyzer, for interpretation of the samples of the outpatient setting.

The fluorescently labeled saliva samples of the outpatient setting were read and documented utilizing the Biotrack-MED® analyzer (patent EP 08874964.3, CE-IVD: NL-CA002-2020-51055, www.biotrack.nl), a semi-autonomous, multi-sample filter cytometer (Biotrack, Leeuwarden, The Netherlands). The Biotrack-MED® analyzer contains a multi-layer neural network that is trained for the interpretation of FISH-based images utilizing the SARS-CoV-2-specific probe, called the SARS-CoV-2 application, which is able to analyze approximately 36 samples per hour. This application produced approximately 150 microscopic fluorescence images (in Z-direction) at a magnification of 200x from which visual recognizable objects were isolated by a computer vision algorithm. Morphometric parameters (n = 20, including shape, area, roundness, brightness and number of spots) are calculated per object [22]. The morphometric parameter scores were subsequently entered in the neural network of the SARS-CoV-2 application, resulting in an autonomous generated test result for SARS-CoV-2 (positive or negative). For a positive result, at least one cell contained 2 or more fluorescent spots. The images generated by the Biotrack-MED® analyzer were blinded and manually analyzed independently by three experienced researchers.

### Statistics

Sensitivity and specificity with 95% confidence intervals were calculated utilizing MedCalc (https://www.medcalc.org/calc/diagnostic_test.php).

## Results

### Proof-of-concept sensitivity and specificity

**Fig 1:**
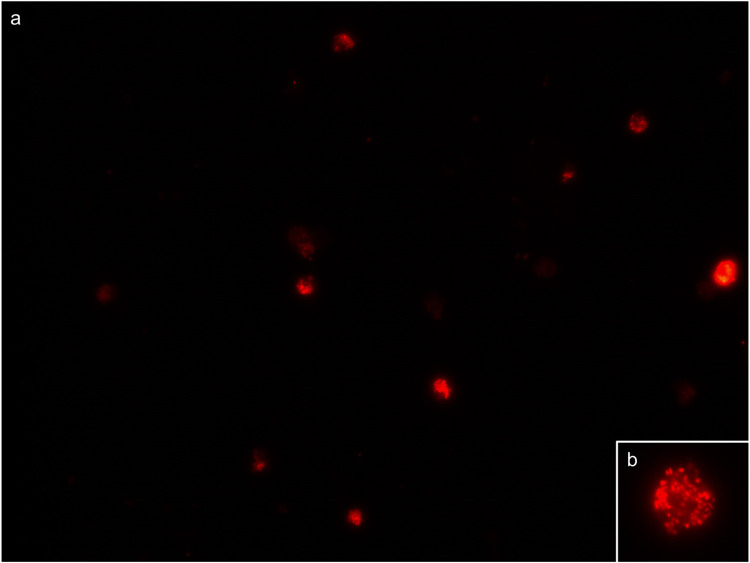
Microscopic image of a positive saliva sample in the proof-of-concept study. Fluorescently-labeled white blood cells (approximately 12–15 μm in size), positive for SARS-CoV-2, are shown at 400x magnification (a). The insert (b) shows a 630x magnification of a white blood cell positive for SARS-CoV-2, showing the highly fluorescent spheres that are typical for SARS-CoV-2 when applying this method.

The fluorescently labeled saliva samples of the proof-of-concept study population were manually analyzed independently by three experienced researchers by means of visual inspection utilizing a fluorescence microscope. A sample was determined positive when multiple highly fluorescent spheres were present in white blood cells (Fig 1) and negative when no fluorescent signal was present. The dataset consisted of 424 samples, of which 100 were RT-PCR positive and 324 were RT-PCR negative for SARS-CoV-2. When analyzing these samples by FISH, 96 were positive, 319 were negative, 5 were false-positive and 4 were false-negative for SARS-CoV-2, compared with RT-PCR outcomes (Tables 4 and 5). This resulted in a sensitivity-score of 96.0% (95% CI 90.0%-98.9%) and a specificity-score of 98.5% (95% CI 96.4%-99.5%). Thus, this FISH-based test and the RT-PCR on nasopharyngeal swab yield comparable results regarding the presence of SARS-CoV-2 in a mild to moderate symptomatic population.

**Table 4:** Results of the saliva samples of the SARS-CoV-2 positive persons.

P	1	2	3	4	5	6	7	8	9	10
1	**P**	**P**	**P**	**P**	**P**	**P**	**P**	**P**	**P**	**P**
2	**P**	**P**	**P**	**P**	**P**	**P**	**P**	**P**	**P**	**P**
3	**P**	**P**	**P**	**P**	**P**	**P**	**P**	**P**	**P**	**P**
4	**P**	***N***	**P**	**P**	**P**	**P**	**P**	**P**	**P**	**P**
5	**P**	**P**	**P**	**P**	**P**	**P**	**P**	**P**	**P**	**P**
6	**P**	**P**	**P**	**P**	**P**	**P**	**P**	**P**	**P**	**P**
7	**P**	**P**	**P**	**P**	**P**	**P**	**P**	**P**	**P**	**P**
8	**P**	***N***	**P**	***N***	**P**	**P**	**P**	**P**	**P**	**P**
9	**P**	**P**	**P**	**P**	**P**	**P**	**P**	**P**	**P**	**P**
10	**P**	**P**	**P**	***N***	**P**	**P**	**P**	**P**	**P**	**P**

P: Positive for SARS-COV-2, N: Negative for SARS-CoV-2

**Table 5:** Results of the saliva samples of the SARS-CoV-2 negative persons.

N	1	2	3	4	5	6	7	8	9	10	11	12	13	14	15	16	17	18
1	**N**	**N**	**N**	**N**	**N**	**N**	**N**	**N**	**N**	**N**	**N**	**N**	**N**	**N**	**N**	**N**	**N**	**N**
2	**N**	**N**	**N**	**N**	**N**	**N**	***P***	**N**	**N**	**N**	**N**	**N**	**N**	**N**	**N**	**N**	**N**	**N**
3	**N**	**N**	**N**	**N**	**N**	**N**	**N**	**N**	**N**	**N**	**N**	**N**	**N**	**N**	**N**	**N**	**N**	**N**
4	**N**	**N**	**N**	**N**	**N**	**N**	**N**	**N**	**N**	**N**	**N**	**N**	**N**	**N**	**N**	**N**	**N**	**N**
5	**N**	**N**	**N**	**N**	**N**	**N**	**N**	**N**	**N**	**N**	**N**	**N**	**N**	**N**	**N**	**N**	**N**	**N**
6	**N**	**N**	**N**	**N**	**N**	**N**	**N**	**N**	**N**	**N**	**N**	**N**	**N**	**N**	**N**	**N**	**N**	**N**
7	**N**	**N**	**N**	**N**	**N**	**N**	**N**	**N**	**N**	**N**	**N**	**N**	**N**	**N**	**N**	**N**	**N**	**N**
8	**N**	**N**	**N**	**N**	**N**	**N**	**N**	**N**	**N**	**N**	**N**	**N**	**N**	**N**	**N**	**N**	**N**	**N**
9	**N**	**N**	**N**	**N**	**N**	**N**	**N**	**N**	**N**	**N**	**N**	**N**	**N**	**N**	**N**	**N**	**N**	**N**
10	***P***	**N**	**N**	**N**	**N**	**N**	**N**	**N**	**N**	**N**	**N**	**N**	**N**	**N**	**N**	**N**	**N**	**N**
11	**N**	**N**	**N**	**N**	**N**	**N**	**N**	**N**	**N**	**N**	**N**	**N**	**N**	**N**	**N**	**N**	**N**	**N**
12	**N**	**N**	**N**	**N**	**N**	**N**	**N**	**N**	**N**	**N**	**N**	**N**	**N**	***P***	**N**	**N**	**N**	**N**
13	**N**	**N**	**N**	**N**	**N**	**N**	**N**	**N**	**N**	**N**	**N**	**N**	**N**	**N**	**N**	**N**	**N**	**N**
14	**N**	**N**	**N**	**N**	**N**	**N**	**N**	**N**	**N**	**N**	**N**	**N**	**N**	**N**	**N**	**N**	**N**	**N**
15	**N**	**N**	**N**	**N**	**N**	**N**	**N**	**N**	**N**	**N**	**N**	**N**	**N**	**N**	**N**	**N**	**N**	***P***
16	**N**	**N**	**N**	***P***	**N**	**N**	**N**	**N**	**N**	**N**	**N**	**N**	**N**	**N**	**N**	**N**	**N**	**N**
17	**N**	**N**	**N**	**N**	**N**	**N**	**N**	**N**	**N**	**N**	**N**	**N**	**N**	**N**	**N**	**N**	**N**	**N**
18	**N**	**N**	**N**	**N**	**N**	**N**	**N**	**N**	**N**	**N**	**N**	**N**	**N**	**N**	**N**	**N**	**N**	**N**

N: Negative for SARS-CoV-2, P: Positive for SARS-COV-2.

### Outpatient sensitivity and specificity

The fluorescently labeled saliva samples of the outpatient study population were analyzed by means of the SARS-CoV-2 application of the Biotrack-MED® analyzer (www.biotrack.nl), an analyzer specifically designed for FISH-based analyses. As visual inspection of microscopic images yielded reliable results comparable to the RT-PCR results in the proof-of-concept dataset, the results of the SARS-CoV-2 application were compared with the visual inspection of the microscopic images created during the analysis for all 972 samples of the outpatient study population.

**Fig 2:**
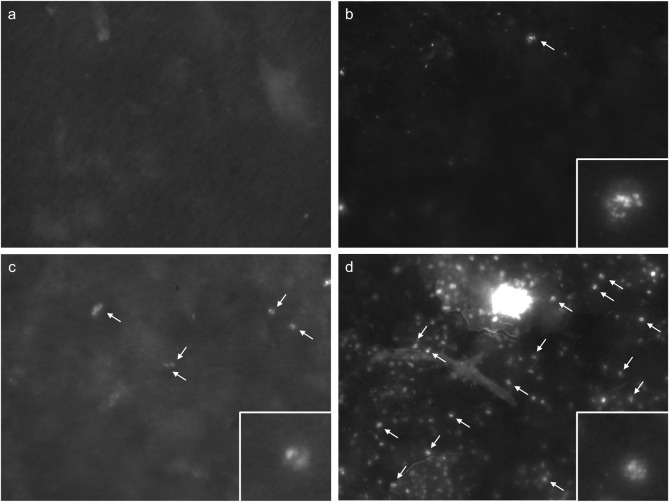
Microscopic images produced by the SARS-CoV-2 application of the Biotrack-MED analyzer in saliva samples. Examples of a negative (a), weak positive (b), medium positive (c) and highly positive (d) test result. The arrows indicate white blood cells positive for SARS-CoV-2 in (b), (c) and (d). In the case of (d), almost all white blood cells appear to show SARS-CoV-2 particles. The images from the Biotrack-MED analyzer are 1392 x 1040 pixels, which converts to 448.9 x 335.4 μm. The insert (b, c and d) shows a positive white blood cell that is zoomed in from the original image. A white blood cell is approximately 12–15 μm in diameter.

The Biotrack-MED® analyzer generated a microscopic fluorescence image for each sample, from which morphometric parameters of the identified microscopic objects were calculated. By means of this information, the SARS-CoV-2 application of the Biotrack-MED® analyzer is able to interpret the microscopic image as being positive or negative for SARS-CoV-2. The saliva samples contained both epithelial cells and white blood cells and the SARS-CoV-2 positive samples showed a variance in the amount of virus-infected white blood cells (Fig 2).

The dataset of the outpatient setting consisted of 972 samples, of which 1 was excluded due to an undisclosed error on the Biotrack-MED® analyzer. When analyzing the remaining 971 samples on the Biotrack-MED® analyzer, applying the SARS-CoV-2 application, 140 were positive, 772 were negative, 45 were false-positive and 14 were false-negative compared to visual inspection (Table 6). This resulted in a sensitivity-score of 90.9% (95% CI 85.2%-94.9%) and a specificity-score of 94.5% (95% CI 92.7%-96.0%). Thus, the SARS-CoV-2 application of the Biotrack-MED® analyzer shows to be valid in the detection of SARS-CoV-2 in an outpatient setting with healthy, mild symptomatic and moderate symptomatic individuals.

**Table 6:** Results of the saliva samples of the SARS-CoV-2 application of the Biotrack-MED® analyzer.

#	Visual	BTMED	#	Visual	BTMED	#	Visual	BTMED
1	**N**	**N**	325	**N**	**N**	649	**N**	**N**
2	**N**	**N**	326	**N**	**N**	650	***P***	**N**
3	**N**	**N**	327	**N**	**N**	651	***P***	***P***
4	**N**	**N**	328	**N**	**N**	652	**N**	**N**
5	**N**	**N**	329	**N**	**N**	653	***P***	***P***
6	**N**	**N**	330	**N**	**N**	654	**N**	**N**
7	**N**	**N**	331	**N**	**N**	655	**N**	**N**
8	**N**	**N**	332	**N**	**N**	656	**N**	**N**
9	**N**	**N**	333	**N**	**N**	657	**N**	***P***
10	**N**	**N**	334	**N**	**N**	658	**N**	**N**
11	**N**	**N**	335	**N**	**N**	659	**N**	**N**
12	**N**	**N**	336	**N**	**N**	660	**N**	**N**
13	**N**	**N**	337	**N**	**N**	661	**N**	**N**
14	**N**	**N**	338	**N**	**N**	662	***P***	***P***
15	**N**	**N**	339	**N**	**N**	663	***P***	***P***
16	**N**	**N**	340	**N**	**N**	664	**N**	**N**
17	**N**	**N**	341	**N**	**N**	665	**N**	**N**
18	**N**	**N**	342	**N**	**N**	666	**N**	**N**
19	**N**	**N**	343	**N**	**N**	667	**N**	**N**
20	**N**	**N**	344	**N**	**N**	668	**N**	**N**
21	**N**	**N**	345	**N**	**N**	669	**N**	**N**
22	**N**	**N**	346	**N**	**N**	670	**N**	**N**
23	**N**	**N**	347	**N**	**N**	671	**N**	**N**
24	**N**	**N**	348	**N**	**N**	672	**N**	**N**
25	**N**	**N**	349	***P***	***P***	673	**N**	**N**
26	***P***	***P***	350	**N**	**N**	674	**N**	**N**
27	**N**	**N**	351	**N**	**N**	675	**N**	**N**
28	**N**	**N**	352	**N**	**N**	676	**N**	**N**
29	**N**	**N**	353	**N**	**N**	677	**N**	**N**
30	**N**	**N**	354	**N**	**N**	678	**N**	**N**
31	**N**	**N**	355	**N**	**N**	679	**N**	**N**
32	**N**	**N**	356	**N**	**N**	680	**N**	**N**
33	**N**	**N**	357	**N**	**N**	681	**N**	**N**
34	***P***	***P***	358	**N**	**N**	682	**N**	**N**
35	**N**	**N**	359	**N**	**N**	683	**N**	**N**
36	**N**	**N**	360	**N**	***P***	684	**N**	**N**
37	**N**	**N**	361	**N**	**N**	685	**N**	**N**
38	**N**	**N**	362	***P***	***P***	686	**N**	**N**
39	**N**	**N**	363	**N**	***P***	687	**N**	**N**
40	**N**	**N**	364	**N**	**N**	688	**N**	**N**
41	**N**	**N**	365	***P***	***P***	689	**N**	**N**
42	**N**	**N**	366	**N**	**N**	690	**N**	**N**
43	**N**	**N**	367	**N**	**N**	691	**N**	**N**
44	***P***	***P***	368	**N**	**N**	692	**N**	**N**
45	**N**	**N**	369	**N**	***P***	693	**N**	**N**
46	**N**	**N**	370	**N**	**N**	694	**N**	**N**
47	**N**	**N**	371	**N**	**N**	695	**N**	**N**
48	**N**	**N**	372	**N**	**N**	696	**N**	**N**
49	**N**	**N**	373	**N**	**N**	697	**N**	**N**
50	**N**	**N**	374	***P***	***P***	698	**N**	**N**
51	**N**	**N**	375	***P***	***P***	699	**N**	**N**
52	**N**	**N**	376	***P***	***P***	700	**N**	**N**
53	**N**	**N**	377	**N**	**N**	701	**N**	**N**
54	**N**	**N**	378	**N**	**N**	702	**N**	**N**
55	**N**	**N**	379	**N**	**N**	703	**N**	**N**
56	**N**	**N**	380	**N**	**N**	704	**N**	**N**
57	**N**	**N**	381	**N**	**N**	705	**N**	***P***
58	**N**	**N**	382	**N**	**N**	706	**N**	***P***
59	**N**	**N**	383	**N**	**N**	707	**N**	**N**
60	**N**	**N**	384	**N**	**N**	708	**N**	**N**
61	**N**	**N**	385	**N**	**N**	709	**N**	**N**
62	**N**	**N**	386	**N**	**N**	710	**N**	**N**
63	**N**	**N**	387	**N**	**N**	711	**N**	**N**
64	**N**	**N**	388	**N**	**N**	712	**N**	**N**
65	**N**	**N**	389	**N**	**N**	713	**N**	**N**
66	**N**	**N**	390	**N**	**N**	714	**N**	**N**
67	**N**	**N**	391	**N**	***P***	715	**N**	**N**
68	**N**	**N**	392	**N**	**N**	716	**N**	**N**
69	**N**	**N**	393	**N**	**N**	717	**N**	**N**
70	**N**	**N**	394	**N**	**N**	718	**N**	**N**
71	**N**	**N**	395	**N**	**N**	719	**N**	**N**
72	**N**	**N**	396	**N**	**N**	720	**N**	**N**
73	***P***	***P***	397	**N**	**N**	721	**N**	**N**
74	***P***	***P***	398	**N**	**N**	722	***P***	**N**
75	***P***	***P***	399	**N**	**N**	723	**N**	**N**
76	***P***	***P***	400	**N**	**N**	724	**N**	**N**
77	***P***	***P***	401	**N**	**N**	725	**N**	**N**
78	***P***	***P***	402	**N**	**N**	726	**N**	**N**
79	***P***	***P***	403	**N**	***P***	727	**N**	**N**
80	**N**	**N**	404	**N**	**N**	728	**N**	**N**
81	***P***	***P***	405	**N**	**N**	729	**N**	***P***
82	***P***	***P***	406	**N**	**N**	730	**N**	***P***
83	***P***	***P***	407	**N**	**N**	731	**N**	**N**
84	***P***	***P***	408	**N**	**N**	732	**N**	**N**
85	***P***	***P***	409	**N**	**N**	733	**N**	**N**
86	***P***	***P***	410	**N**	**N**	734	**N**	**N**
87	***P***	***P***	411	**N**	**N**	735	***P***	***P***
88	***P***	***P***	412	**N**	***P***	736	***P***	***P***
89	***P***	***P***	413	**N**	**N**	737	**N**	**N**
90	**N**	**N**	414	**N**	**N**	738	**N**	**N**
91	***P***	***P***	415	**N**	**N**	739	**N**	**N**
92	***P***	***P***	416	**N**	**N**	740	**N**	**N**
93	***P***	***P***	417	**N**	**N**	741	**N**	**N**
94	***P***	**N**	418	**N**	**N**	742	**N**	**N**
95	***P***	***P***	419	**N**	**N**	743	**N**	**N**
96	***P***	***P***	420	**N**	**N**	744	**N**	**N**
97	***P***	***P***	421	**N**	***P***	745	**N**	**N**
98	***P***	**N**	422	**N**	**N**	746	***P***	***P***
99	***P***	ERROR	423	**N**	**N**	747	***P***	***P***
100	**N**	**N**	424	**N**	**N**	748	**N**	**N**
101	***P***	***P***	425	**N**	***P***	749	**N**	**N**
102	***P***	**N**	426	**N**	**N**	750	**N**	**N**
103	***P***	***P***	427	**N**	**N**	751	**N**	**N**
104	***P***	***P***	428	**N**	**N**	752	**N**	**N**
105	***P***	***P***	429	**N**	**N**	753	**N**	***P***
106	***P***	***P***	430	**N**	**N**	754	**N**	**N**
107	***P***	***P***	431	**N**	**N**	755	**N**	**N**
108	**N**	**N**	432	**N**	**N**	756	**N**	**N**
109	**N**	**N**	433	**N**	**N**	757	**N**	**N**
110	**N**	**N**	434	**N**	**N**	758	**N**	**N**
111	**N**	**N**	435	**N**	**N**	759	**N**	**N**
112	**N**	**N**	436	***P***	***P***	760	**N**	**N**
113	**N**	**N**	437	***P***	***P***	761	**N**	**N**
114	**N**	**N**	438	**N**	**N**	762	**N**	**N**
115	**N**	**N**	439	**N**	**N**	763	**N**	**N**
116	**N**	**N**	440	***P***	***P***	764	**N**	**N**
117	**N**	**N**	441	**N**	***P***	765	**N**	***P***
118	**N**	**N**	442	**N**	**N**	766	**N**	**N**
119	**N**	**N**	443	**N**	***P***	767	**N**	**N**
120	**N**	**N**	444	**N**	**N**	768	**N**	**N**
121	**N**	**N**	445	**N**	**N**	769	**N**	**N**
122	**N**	**N**	446	***P***	***P***	770	**N**	**N**
123	**N**	**N**	447	***P***	***P***	771	**N**	**N**
124	**N**	**N**	448	***P***	***P***	772	**N**	**N**
125	**N**	**N**	449	**N**	**N**	773	**N**	**N**
126	**N**	**N**	450	**N**	**N**	774	**N**	**N**
127	**N**	**N**	451	**N**	**N**	775	**N**	**N**
128	**N**	**N**	452	**N**	**N**	776	**N**	**N**
129	**N**	**N**	453	**N**	**N**	777	**N**	**N**
130	**N**	**N**	454	**N**	**N**	778	**N**	**N**
131	**N**	**N**	455	**N**	**N**	779	**N**	***P***
132	**N**	**N**	456	**N**	**N**	780	**N**	**N**
133	**N**	**N**	457	**N**	**N**	781	**N**	**N**
134	**N**	**N**	458	**N**	**N**	782	**N**	**N**
135	**N**	**N**	459	**N**	**N**	783	**N**	**N**
136	**N**	**N**	460	**N**	**N**	784	**N**	**N**
137	**N**	***P***	461	**N**	**N**	785	***P***	***P***
138	**N**	**N**	462	***P***	***P***	786	**N**	**N**
139	**N**	**N**	463	**N**	**N**	787	**N**	**N**
140	**N**	**N**	464	**N**	**N**	788	**N**	**N**
141	**N**	**N**	465	**N**	**N**	789	**N**	**N**
142	**N**	**N**	466	**N**	**N**	790	**N**	**N**
143	**N**	**N**	467	**N**	**N**	791	**N**	**N**
144	**N**	**N**	468	**N**	**N**	792	**N**	**N**
145	***P***	***P***	469	**N**	**N**	793	**N**	**N**
146	**N**	**N**	470	**N**	**N**	794	**N**	**N**
147	***P***	***P***	471	**N**	**N**	795	**N**	**N**
148	***P***	***P***	472	**N**	**N**	796	***P***	***P***
149	***P***	***P***	473	**N**	**N**	797	***P***	**N**
150	***P***	***P***	474	**N**	***P***	798	**N**	**N**
151	***P***	***P***	475	**N**	**N**	799	**N**	**N**
152	**N**	**N**	476	**N**	**N**	800	**N**	**N**
153	***P***	***P***	477	**N**	**N**	801	**N**	**N**
154	***P***	***P***	478	**N**	**N**	802	**N**	**N**
155	***P***	***P***	479	**N**	***P***	803	**N**	**N**
156	***P***	***P***	480	**N**	**N**	804	**N**	**N**
157	**N**	**N**	481	**N**	**N**	805	**N**	**N**
158	**N**	**N**	482	**N**	**N**	806	***P***	**N**
159	***P***	***P***	483	**N**	**N**	807	***P***	***P***
160	**N**	**N**	484	**N**	***P***	808	***P***	***P***
161	***P***	***P***	485	**N**	**N**	809	**N**	**N**
162	***P***	***P***	486	**N**	**N**	810	**N**	**N**
163	***P***	***P***	487	**N**	**N**	811	**N**	**N**
164	P	P	488	N	N	812	N	N
165	P	N	489	N	N	813	N	N
166	P	P	490	N	N	814	N	N
167	P	P	491	N	N	815	N	N
168	N	N	492	N	N	816	N	N
169	P	P	493	N	N	817	N	N
170	P	P	494	N	N	818	N	P
171	P	P	495	N	N	819	N	N
172	P	P	496	N	N	820	N	N
173	P	P	497	N	P	821	N	N
174	P	P	498	N	N	822	N	N
175	P	P	499	N	N	823	N	N
176	P	P	500	N	P	824	N	N
177	P	P	501	N	N	825	N	P
178	P	P	502	N	N	826	N	N
179	P	P	503	N	N	827	N	N
180	N	N	504	N	N	828	N	N
181	N	N	505	N	N	829	N	N
182	N	N	506	N	N	830	N	N
183	N	N	507	N	N	821	N	N
184	N	N	508	P	P	832	N	N
185	N	N	509	P	P	833	N	N
186	N	N	510	N	N	834	N	N
187	N	N	511	P	P	835	N	N
188	N	N	512	N	N	836	N	N
189	N	N	513	N	P	837	N	N
190	N	N	514	N	N	838	N	N
191	N	N	515	N	P	839	N	N
192	N	N	516	N	N	840	N	N
193	N	N	517	N	N	841	N	N
194	N	N	518	P	P	842	N	N
195	N	N	519	P	P	843	N	N
196	N	N	520	P	P	844	N	N
197	N	N	521	N	N	845	N	N
198	N	N	522	N	N	846	N	N
199	N	N	523	N	N	847	N	N
200	N	N	524	N	N	848	N	N
201	N	N	525	N	N	849	N	N
202	N	N	526	N	P	850	N	N
203	N	N	527	N	N	851	P	N
204	N	N	528	N	N	852	N	N
205	N	N	529	N	N	853	N	N
206	N	N	530	N	N	854	N	N
207	N	N	531	N	N	855	N	N
208	N	N	532	N	N	856	N	N
209	N	N	533	N	N	857	N	N
210	N	N	534	N	N	858	N	N
211	N	N	535	N	N	859	N	P
212	N	N	536	N	N	860	N	N
213	N	N	537	N	N	861	N	N
214	N	N	538	N	N	862	N	N
215	N	N	539	N	N	863	N	N
216	N	N	540	N	N	864	N	N
217	P	P	541	N	N	865	N	N
218	P	P	542	N	P	866	N	N
219	P	P	543	N	N	867	N	N
220	P	P	544	N	N	868	N	N
221	P	P	545	N	N	869	P	P
222	P	P	546	N	N	870	N	N
223	P	P	547	N	N	871	P	N
224	P	N	548	N	N	872	N	N
225	P	P	549	N	N	873	P	P
226	P	N	550	N	N	874	N	N
227	P	P	551	N	N	875	N	P
228	P	P	552	N	N	876	N	N
229	N	P	553	N	N	877	N	N
230	P	P	554	N	N	878	P	P
231	P	P	555	N	N	879	P	P
232	P	P	556	N	P	880	P	P
233	P	P	557	N	N	881	N	P
234	N	N	558	N	N	882	N	N
235	P	P	559	N	N	883	P	P
236	P	P	560	N	N	884	N	N
237	P	P	561	N	N	885	N	N
238	N	P	562	N	N	886	N	N
239	P	P	563	N	N	887	N	N
240	P	P	564	N	N	888	N	N
241	P	P	565	N	P	889	N	N
242	P	P	566	N	N	890	N	N
243	P	P	567	N	N	891	N	N
244	N	N	568	N	N	892	N	N
245	P	P	569	N	N	893	N	N
246	N	N	570	N	P	894	N	N
247	P	P	571	N	N	895	N	N
248	P	P	572	N	N	896	N	N
249	P	P	573	N	N	897	N	N
250	N	N	574	N	N	898	N	N
251	P	P	575	N	N	899	N	N
252	N	N	576	N	N	900	N	N
253	N	N	577	N	N	901	N	N
254	N	N	578	N	N	902	N	N
255	N	N	579	N	N	903	N	N
256	N	N	580	P	P	904	N	N
257	N	N	581	P	P	905	N	N
258	N	N	582	N	N	906	N	N
259	N	N	583	P	P	907	N	N
260	N	N	584	N	N	908	N	N
261	N	N	585	P	P	909	N	N
262	N	N	586	N	N	910	N	N
263	N	N	587	N	N	911	N	N
264	N	N	588	N	N	912	N	N
265	N	N	589	N	N	913	N	N
266	N	N	590	P	P	914	N	N
267	N	N	591	P	P	915	N	N
268	N	P	592	P	P	916	N	N
269	N	N	593	N	N	917	N	N
270	N	N	594	N	N	918	N	N
271	N	N	595	N	N	919	N	N
272	N	N	596	N	P	920	N	N
273	N	N	597	N	N	921	N	N
274	**N**	**N**	598	**N**	**N**	922	**N**	***P***
275	**N**	**N**	599	**N**	**N**	923	**N**	**N**
276	**N**	**N**	600	**N**	**N**	924	**N**	**N**
277	**N**	**N**	601	**N**	**N**	925	**N**	**N**
278	**N**	**N**	602	**N**	**N**	926	**N**	**N**
279	**N**	**N**	603	**N**	**N**	927	**N**	**N**
280	**N**	**N**	604	**N**	**N**	928	**N**	**N**
281	**N**	**N**	605	**N**	**N**	929	**N**	**N**
282	**N**	**N**	606	**N**	**N**	930	**N**	**N**
283	**N**	**N**	607	**N**	**N**	931	**N**	**N**
284	**N**	**N**	608	**N**	**N**	932	**N**	**N**
285	**N**	**N**	609	**N**	**N**	933	**N**	**N**
286	**N**	**N**	610	**N**	**N**	934	**N**	**N**
287	**N**	**N**	611	**N**	**N**	935	**N**	**N**
288	**N**	**N**	612	**N**	**N**	936	**N**	**N**
289	**N**	**N**	613	**N**	**N**	937	**N**	**N**
290	**N**	**N**	614	**N**	**N**	938	**N**	**N**
291	**N**	**N**	615	**N**	**N**	939	**N**	**N**
292	***P***	**N**	616	**N**	**N**	940	**N**	**N**
293	**N**	**N**	617	**N**	**N**	941	***P***	***P***
294	**N**	**N**	618	**N**	**N**	942	**N**	**N**
295	***P***	***P***	619	**N**	**N**	943	**N**	**N**
296	**N**	**N**	620	**N**	**N**	944	**N**	**N**
297	**N**	***P***	621	**N**	**N**	945	**N**	***P***
298	**N**	**N**	622	**N**	**N**	946	**N**	**N**
299	**N**	**N**	623	**N**	**N**	947	**N**	**N**
300	***P***	***P***	624	**N**	**N**	948	**N**	**N**
301	**N**	**N**	625	**N**	**N**	949	**N**	**N**
302	***P***	***P***	626	**N**	**N**	950	***P***	***P***
303	***P***	***P***	627	**N**	**N**	951	***P***	***P***
304	**N**	**N**	628	**N**	**N**	952	***P***	***P***
305	***P***	***P***	629	**N**	**N**	953	**N**	**N**
306	**N**	**N**	630	**N**	**N**	954	**N**	**N**
307	**N**	**N**	631	**N**	**N**	955	**N**	**N**
308	**N**	**N**	632	**N**	**N**	956	**N**	**N**
309	**N**	**N**	633	**N**	**N**	957	**N**	**N**
310	**N**	**N**	634	**N**	**N**	958	**N**	**N**
311	**N**	**N**	635	**N**	**N**	959	**N**	**N**
312	**N**	**N**	636	**N**	**N**	960	**N**	**N**
313	**N**	**N**	637	**N**	**N**	961	**N**	**N**
314	**N**	**N**	638	**N**	**N**	962	**N**	**N**
315	**N**	**N**	639	**N**	**N**	963	**N**	**N**
316	**N**	**N**	640	**N**	**N**	964	**N**	**N**
317	**N**	**N**	641	**N**	**N**	965	**N**	**N**
318	***P***	***P***	642	**N**	**N**	966	**N**	**N**
319	**N**	**N**	643	**N**	**N**	967	**N**	**N**
320	**N**	**N**	644	**N**	**N**	968	**N**	**N**
321	**N**	***P***	645	**N**	***P***	969	**N**	**N**
322	**N**	**N**	646	**N**	**N**	970	**N**	**N**
323	**N**	**N**	647	**N**	**N**	971	**N**	**N**
324	**N**	**N**	648	***P***	**N**	972	**N**	**N**

N: Negative for SARS-CoV-2, P: Positive for SARS-COV-2.

## Discussion

In this study, a FISH-based method to detect SARS-CoV-2 in saliva was first evaluated in a proof-of-concept setting. It was shown that this test has a high sensitivity (96.0%) and specificity (98.5%) when analyzing 100 RT-PCR positive and 324 RT-PCR negative saliva samples, which is according to the WHO guidelines (at least 100 RT-PCR positive and at least 300 RT-PCR negative samples for the method to be tested) [23]. Thereafter, the applicability of the method was tested in an outpatient setting utilizing the Biotrack-MED® analyzer, a reading platform specific for FISH-bases analyses. This resulted in a sensitivity of 90.9% and a specificity of 94.5%. Therefore we conclude that the FISH-based SARS-CoV-2 application of the Biotrack-MED® analyzer is a sensitive and reliable assay for the detection of SARS-CoV-2 in the general population.

FISH is an easy to use analytical method with a high sensitivity to detect specific RNA or DNA sequences and a relatively short time to result. It is a reliable technique that has been optimized in its 20+ years of existence [24,25]. Moreover, FISH has proven to be a valid an practical strategy in the direct *in situ* detection of SARS-CoV-2, utilizing SARS-CoV-2-specific and fluorescently-labeled oligonucleotide probes [26]. In the current evaluation study, a SARS-CoV-2-specific probe, directed at the nucleocapsid (N) gene, is utilized. This specific probe shows *in silico* no cross-reactivity with other viruses that may be present in saliva, such as influenza virus or rhinovirus. Using this N-gene-specific probe, SARS-CoV-2 infection in different disease states (mild to moderate) and asymptomatic persons could be detected. As also small amounts of virus could be detected (LoD of 1.4 copies of viral RNA per 1 μl sample), this FISH-based method shows a similar detection level in the early infections phases as compared with the RT-PCR. The current study did not examine whether a correlation existed between the viral load of the RT-PCR and the FISH-based method. This query is in this study not relevant, as the main aim was to evaluate the FISH-based method and whether it could detect an infected person.

The SARS-CoV-2 genomic RNA is single-stranded and has a sense orientation. During the replication of SARS-CoV-2, intermediate RNAs with an antisense orientation are formed [27]. The SARS-CoV-2 probe (COV19 Probe) utilized in this study is only binding to the SARS-CoV-2 intermediate RNA with the antisense orientation of the nucleocapsid gene. Therefore, the SARS-CoV-2 application described in this study detects SARS-CoV-2 when it is replicating and, thus, ‘active’ [19]. The RT-PCR is able to detect both intra- and extracellular sense genomic and antisense intermediate RNAs, although both techniques are able to detect SARS-CoV-2 presence, the SARS-CoV-2 application described here detects only the ‘active’ virus, the intermediate RNAs, while the RT-PCR also may only detect the genomic RNA, which gives information about the presence of the virus, but not of its replicative stage. This is confirmed by studies showing that SARS-CoV-2 could be detected by RT-PCR of nasopharyngeal swabs more than 21 days after disease onset [12]. In addition, the SARS-CoV-2 application detects only the intracellular virus (in white blood cells) and not the extracellular virus (free virions in the saliva). As the cells are the replication site for SARS-CoV-2, the use of the FISH-based SARS-CoV-2 application may yield (diagnostic) information about the viral activity.

The FISH-based method described in this study resulted in approximately 5% false positive and approximately 9% false negative results. The false positive results were most often due to contamination during sampling. For example, small food remnants may be mistaken by the SARS-CoV-2 application as positive white blood cells when the morphometrics are similar. However, a percentage false positive and false negative results are present in all diagnostic tests. In the case of the RT-PCR, percentages up to 6.9% and 58% were reported for false positive and false negative results, respectively [28–30]. The resulting sensitivity and specificity of the outpatient setting in this study are 90.9% and 94.5%, respectively. When comparing with the current ‘gold standard’ for SARS-CoV-2 detection, the RT-PCR, these percentages are comparable or higher than the RT-PCR, where the sensitivity ranges between 32–93% [30–33]. Thus, this FISH-based method in combination with the SARS-CoV-2 application of the Biotrack-MED® analyzer show comparable false positives and false negatives to the RT-PCR, which suggest it to be a reliable assay for the detection of SARS-CoV-2.

The matrix of choice in this study is saliva. For SARS-CoV-2 to enter and infect a cell, it must bind to ACE2 receptors. These receptors are found in the oral cavity, with (high) expression in the epithelial cells of the tongue and epithelial cells, T cells, B cells and fibroblasts of the oral mucosa [13]. In an early stage of the infection, the virus may only be present in the salivary glands, and from this reservoir the infection may spread throughout the body. This may result in the presence of SARS-CoV-2 in saliva in (very) early stages, while the virus is not yet present in the nose and/or throat, rendering the RT-PCR negative [10,34]. The SARS-CoV-2 presence in saliva is the highest around the time of symptom onset [13]. However, SARS-CoV-2 can also be detected in saliva in asymptomatic individuals [15]. As saliva is a matrix that is easily dispersed (in the form of droplets or aerosols, which are able to survive as long as 90 minutes) by a SARS-CoV-2 patient, persons in the vicinity may get infected by inhaling the droplets and/or aerosols or by bringing the droplets to the mouth, nose or eyes via the hands. This may occur also when the patients has no (asymptomatic) or (very) mild symptoms [13,14,34]. As described above, the probe utilized in this study only detects the ‘active’ virus, i.e. the virus in its replicative state. Therefore, this method may also be indicative of the contagiousness of the infected person. Further research should verify this latter statement. However, studies showed that RT-PCR on nasopharyngeal swabs were 20–30% more positive more than 21 days after disease onset compared to RT-PCR on saliva, suggesting that nasopharyngeal swabs detected historical infections, while saliva is more prone to detect an active infection [12]. In addition, other studies reported that there was less variation in SARS-CoV-2 RNA levels in saliva compared to a nasopharyngeal swab during the course of the infection [14,15]. This information is beneficial for both the economy and mental health as it may prevent unnecessarily long isolation periods of infected individuals.

An advantage of utilizing saliva instead of a nasopharyngeal swab is the easy and non-invasive means of sample collection. This also makes the evaluated method better applicable for children. However, there are some discrepancies between different studies comparing RT-PCR on nasopharyngeal swab and saliva. These discrepancies may be explained by differences in utilized protocols, type of saliva, variation in nasopharyngeal sampling, processing of samples, sensitivity of the utilized tests [14,15,35]. The saliva samples used in the current study are collected in sample collection tubes containing Biotrack Preservation Fluid, which fixates the saliva sample within 15 minutes after production, thereby rendering the possible present virus particles harmless for the technician handling the sample afterwards. In addition, due to the immediate fixation procedure upon collection, variables such as temperature, biological degradation and storage time are minimized. Moreover, as the person rinses his/her mouth before the collection of saliva, no influences of food and/or drinks are present.

A limitation of this study is the small amount of included persons in the proof-of-concept setting. For most of the persons that used the commercial services of Biotrack, Leeuwarden to detect the presence of SARS-CoV-2 in their saliva between 1-10-2020 and 1-12-2020, there was no information about whether the person did also a RT-PCR and/or the result of the RT-PCR. Of 28 persons, the result of the RT-PCR was known. Unfortunately, as the RT-PCR was performed by local Dutch Municipal Health Services, the result given was only negative or positive, without Ct-values. As 28 samples are too few to use for machine learning, this increases greatly the chance of overfitting. Therefore, we pooled the samples, to increase the dataset and reduce the change of overfitting in the machine learning of the SARS-CoV-2 application of the Biotrack-MED® analyzer.

*In silico* research showed no cross-reactivity of the COV19 Prove with other viruses that may be present in saliva, such as influenza virus or rhinovirus. However, this should also be tested *in vitro*. Nevertheless, in the winter 2020/2021 hardly any other viruses than SARS-CoV-2 were measured in the Dutch population [36], thus the positive results in this study are due to a SARS-CoV-2 infection.

Another limitation of this study is the inability of the COV19 Probe to distinguish between the different mutant strains of SARS-CoV-2, including the alpha-, beta- and omicron-variant. Although the utilized probe is not able to make a distinction between the strains, it is able to detect all the strains, both *in silico* and *in vitro*.

In conclusion, the evaluation of saliva samples demonstrated that the FISH-based SARS-CoV-2 application of the Biotrack-MED® analyzer is a reliable assay for detection of SARS-CoV-2 infection in an outpatient setting with healthy, mild-symptomatic and moderate-symptomatic individuals.
